# Misinformation reminders enhance belief updating and memory for corrections: the role of attention during encoding revealed by eye tracking

**DOI:** 10.1186/s41235-025-00649-y

**Published:** 2025-07-06

**Authors:** Bayley M. Wellons, Christopher N. Wahlheim

**Affiliations:** Department of Psychology, University of North Carolina at Greensboro, 296 Eberhart Building, P. O. Box 26170, Greensboro, NC 27402-6170 USA

**Keywords:** Attention, Eye fixations, Belief updating, Misinformation reminders, Misinformation correction, Recollection

## Abstract

Misinformation exposure can cause inaccurate beliefs and memories. These unwanted outcomes can be mitigated when misinformation reminders—veracity-labeled statements that repeat earlier-read false information—appear before corrections with true information. The present experiment used eye tracking to examine the role of attention while encoding corrective details in the beneficial effects of reminder-based corrections. Participants read headlines in a belief-updating task that included a within-subjects manipulation of correction format. They first rated the familiarity and veracity of true and false headlines (Phase 1). Then, they read true headlines that corrected false headlines or affirmed true headlines (Phase 2). The true headlines appeared (1) without veracity labels, (2) with veracity labels, or (3) with misinformation reminders and veracity labels. Finally, participants re-rated the veracity of the Phase 1 headlines and rated their memory for whether those headlines were corrected in Phase 2 (Phase 3). Reminder-based corrections led to the greatest reduction in false beliefs, best high confidence recognition of corrections, and earliest eye fixations to the true details of corrections during encoding in Phase 2. Corrections remembered with the highest confidence rating were associated with more and earlier fixations to true details in correction statements in Phase 2. Collectively, these results suggest that misinformation reminders directed attention to corrective details, which improved encoding and subsequent memory for veracity information. These results have applied implications in suggesting that optimal correction formats should include features that direct attention to, and thus support encoding of, the contrast between false and true information.

Exposure to misinformation on the internet can lead to inaccurate beliefs and memories that have negative societal consequences. For example, misinformation about the COVID-19 virus shared on social media platforms reduced vaccination willingness and health guideline compliance (Roozenbeek et al., 2020). These effects may be mitigated by issuing corrections after misinformation exposure, which is generally effective across contexts (Porter & Wood, 2024). Although experts urged against repeating misinformation during corrections because repetition increases perceived truth (for a review, see Lewandowsky et al., 2012), corrections with misinformation reminders enhance the accuracy of inferences, memories, and beliefs (Ecker et al., 2017; Kemp et al., 2022a, 2022b, 2024b; Wahlheim et al., 2020, 2025). A key insight from this work is that misinformation reminders reduce false beliefs by improving memory for corrections. Here, we characterized another aspect of cognition that contributes to these effects: Using eye tracking, we show that reminders direct attention to corrective details, thereby improving memory and belief accuracy.

## Repetition-induced truth and misinformation corrections

Misinformation masquerading as true information affects memory and reasoning even after it has been corrected. This undesirable proactive effect of memory is referred to as the continued influence effect (Johnson & Seifert, 1994; Wilkes & Leatherbarrow, 1988). Initially, there was concern that repeating misinformation during corrections would exacerbate the continued influence effect by making misinformation more familiar and fluent, and thus more believable (for a review, see Lewandowsky et al., 2012). The idea that familiarity and the perceived veracity of misinformation are associated in this way is supported by studies showing that repetition increases the perceived veracity of information regardless of its actual veracity or plausibility (e.g., Fazio et al., 2019; Hasher et al., 1977; Pennycook et al., 2018). Repetition-induced perceptions of truth beget the concern that repeating misinformation in corrections could backfire by increasing its familiarity and, thus, perceived veracity. According to dual-process models, familiarity backfire should be most likely under conditions that impair recollection of contextual information (Begg et al., 1992; Skurnik et al., 2005), such as when response competition is heightened by presenting false and true information in close temporal proximity. However, studies have failed to replicate the familiarity backfire effect (e.g., Cameron et al., 2013; Swire et al., 2017) and have attributed it to poor test–retest reliability and inappropriate interpretations of constructs (for reviews, see Swire-Thompson et al., 2020, 2022).

Findings from a collection of studies have led to an emerging consensus that reminding people of misinformation while providing corrective details can improve subsequent veracity perceptions and memory for corrections. In experimental tasks, misinformation reminders are statements that repeat previously encountered false details with added contextual information indicating that the details are incorrect. This context often consists of a label indicating that the information is false and an immediately subsequent presentation of true, corrective details including a veracity label (see Kemp et al., 2024a). The re-presentation of false details is presumed to trigger remindings of prior encounters that enhance the elaborative encoding that occurs when the false and true details are contrasted (Wahlheim et al., 2020). These reminder effects are measured via belief accuracy (i.e., lower belief ratings for false information and higher belief ratings for true information) following corrections. A foundational test of reminder effects on beliefs (indirectly assessed via inferential reasoning) was conducted using a narrative-based continued influence effect paradigm (Ecker et al., 2017). Participants read stories containing false details that were later corrected, with corrections varying in the extent to which they included details that referenced the misinformation. Misinformation had the weakest effect on inferences following complete reminders compared to partial or no reminders.

These results are consistent with the integrative encoding account of belief and memory updating. According to this view, co-activation of false and true information increases conflict saliency by upregulating attention, which can improve subsequent memory (Ecker et al., 2017; Kendeou et al., 2014). Simultaneous activation of false and true details facilitates integration (associative encoding) of these details and their veracity. This was shown by an experiment requiring participants to think aloud while reading refutational texts (Kendeou et al., 2019). Participants’ statements indicated metacognitive monitoring of conflict when false and true information were simultaneously accessible in consciousness. Confusion and comprehension monitoring decreased after reading the critical sentence in the refutation text. This is consistent with the idea that integrated information resolves competition between false and true information and promotes comprehension. These results suggest that repeating false information during corrections facilitates integration of true and false information and memory for its veracity.

Following these studies, Wahlheim et al. (2020) examined the effects of including reminders of everyday misinformation from the internet with corrections in news headline formats on memory and belief accuracy. Their guiding framework was a dual-process approach including roles for familiarity, integrative encoding, and recollection of corrections. According to this *memory-for-change framework* (Wahlheim & Jacoby, 2013), reminders should increase the accessibility of misinformation and allow for integrative encoding that promotes subsequent recollection of corrections. Conversely, failed integration should lead to recollection failure that allows misinformation familiarity to reduce memory and belief accuracy. Several studies of misinformation reminder effects on memory and beliefs support this account.

In a study closely following Ecker et al. (2017), participants read headlines with true and false information of unclear veracity from fact-checking websites (Wahlheim et al., 2020). Next, two types of headlines with true details that corrected false details appeared (along with control conditions): Veracity-labeled true headlines appeared after veracity-labeled reminders of false headlines from the prior phase, and unlabeled true headlines appeared alone. On a subsequent test, corrections with misinformation reminders improved cued recall of true and false details and memory that corrections occurred. Corrections with reminders and veracity labels also increased the perceived veracity of recalled true information and decreased the perceived veracity of intrusions of false information. These improvements in belief accuracy occurred most for reminder-based corrections. Another study replicated these effects with updated stimuli in an experiment that compared corrections with reminders and veracity labels to only corrections with veracity labels (Kemp et al., 2022b). That study found that reminders improved recall of true information and memory for corrections the most. This ruled out the alternative explanation that reminder benefits on memory accuracy reflected only the enhanced conflict saliency from labeling true headlines. However, one limitation of these studies is that the belief measures only assessed the perceived veracity of details reported after attempts at recalling true information.

To determine how reminders affect false beliefs across all corrected headlines, another study used headlines resembling social media posts in a task measuring beliefs before and after corrections (Wahlheim et al., 2025). Participants first rated the veracity of true and false headlines; then, three types of veracity-labeled corrections appeared: only reminders of false headlines, only corrections showing true headlines, and a combination of two—reminders of false headlines shown before corrections with true headlines. Two more phases occurred immediately and after a delay (1 week or 1 month). Participants re-rated the veracity of all headlines and rated their memory for headlines being corrected. Corrections with reminders before true details reduced false beliefs and improved memory for corrections the most. False belief reduction was also greater in all conditions when corrections were confidently recognized. This rating was assumed to be the most sensitive to recollection-based retrieval, which should include contextual information that a headline was corrected (e.g., remembering contrasting veracity labels). Such contextual details should support confident memory for corrections, but higher confidence was not assumed to always reflect recollection-based retrieval because no measure is process pure (Jacoby, 1991). Indeed, these ratings can also reflect false recollections or strong feelings of familiarity. Collectively, these results suggest that contrasting false and true information promoted integrative encoding that supported memory for corrections, which in turn accurately informed perceptions of headline veracity. Given that conscious attention promotes subsequent memory (for reviews, see Long et al., 2018; Sherman & Turk-Browne, 2022), misinformation reminders may have exerted their effects by guiding attention to true details.

## Attention, memory, and eye tracking

Measuring eye fixations on corrective details can determine if reminders upregulate attention to those details. Fixations are periods when the eyes remain nearly motionless on a portion of a stimulus (King et al., 2019). Measuring fixations is an established method for evaluating the orientation and level of attention during stimulus viewing as indicated by its precision and associations with memory (Loftus, 1972; Tversky, 1974; Vraga et al., 2016). Although viewers can also shift attention covertly (i.e., without changing gaze location), fixations directly indicate the object of overt attention (Just & Carpenter, 1976; Vraga et al., 2016) and are associated with successful memory (e.g., Bylinskii et al., 2015; Damiano & Walther, 2019; Olejarczyk et al., 2014). These characteristics make fixations ideal for identifying the features that receive the most attention during encoding (Duchowski, 2007; King et al., 2019). Differences in fixations to corrective details and their associations with memory could reveal how correction features in prior studies attracted attention during encoding.

Memory-guided attention is also reflected in eye movements. This was shown, for example, when participants reinstated fixation patterns from encoding while recognizing repeated stimuli (Noton & Stark, 1971). Research has replicated this result and shown that memory directs attention and attendant fixations (for a review, see Hannula et al., 2010). For example, Ryan et al. (2007) compared gaze patterns for novel and previously seen faces. Distinct fixation patterns for repeated faces emerged independently of instructions to view only one face category. This suggested that viewers’ memory for repeated faces directed attention to compare memories of old faces with current perceptions. This result replicated in a study comparing eye movements for repeated and similar novel faces (Hannula et al., 2012). Differential fixation patterns were observed within the first few seconds of face viewing, which implicated a preference for repeated over similar faces. Disproportionate viewing of repeated faces occurred regardless of the similarity between repeated and novel faces and emerged before responses to a behavioral measure of face memory, which suggested that memory inherently influenced eye movements. Memory guidance has also been shown in studies of attention to anticipated locations (e.g., Boettcher et al., 2022; Hollingworth, 2009; Huestegge & Koch, 2012). Collectively, these findings suggest that retrieved memories guide attention, thus enabling comparison between existing memories and current perceptions, which, in the present study, may support memory for headline veracity and whether corrections occurred.

Anticipatory fixations also evince the role of memory-guided attention in encoding feature changes, such as those present in misinformation corrections. Viewers fixate on the locations where earlier-viewed information appeared (e.g., Johansson & Johansson, 2014), which can reinstate the earlier-viewed information and its spatiotemporal context (Kragel & Voss, 2021; Wynn et al., 2019). Viewers should thus be more likely to notice and encode changes if they attend to these locations, which can lead to updated memories. This is consistent with the proposal that reminders of false information direct attention to true details. Experiments on memory updating for observed actions indirectly supported this proposal (Wahlheim et al., 2022). In that study, viewers watched movies of an actor contacting related objects in adjacent screen locations across two movies (e.g., unlocking a deadbolt vs. door handle). Memory for recent actions was better when participants remembered that those actions changed. This memory advantage was associated with more fixations to the first contact location while viewers anticipated the second action. The association between predictive looking and memory indicates that fixations assayed viewers’ memory-guided attention to the stimulus features of retrieved memories and perceptual inputs. This anticipatory looking pattern suggests that memory-guided attention evoked by reminder-based corrections may lead to earlier looking to corrective details. 

### The present study

While some studies have used eye tracking to identify features that draw attention to news stories in social media contexts (e.g., Sülflow et al., 2019; Vraga et al., 2016), eye tracking has not been used to investigate how reminders of false headlines in social media formats guide attention to veracity changes and how such attention associates with beliefs and memory. We addressed this gap here. Our guiding hypothesis was that reminding people of false details just before presenting true details should best stimulate memory-guided attention to the true details and subsequent recognition that false headlines were corrected. Based on prior findings, we predicted that participants would show the most accurate beliefs in false headlines (viz. lowest perceived veracity ratings) in association with the most confident self-reported memories that false details were corrected, referred to as recognition of corrections (Wahlheim et al., 2025).

The procedure used a within-subjects design that included three phases. In the initial evaluation phase (Phase 1), participants rated the preexisting familiarity and veracity of true and false headlines of unclear veracity. In the correction phase (Phase 2), false headlines from the initial evaluation phase were corrected using three formats. Some true headlines appeared without veracity labels (unlabeled true headlines), other true headlines appeared with veracity labels (labeled true headlines), and a final set of true headlines appeared with veracity labels just after reminders of false headlines that also included veracity labels (labeled false and true headlines). So that participants were required to detect the unlabeled true headlines that contradicted/corrected false information, true headlines from the initial evaluation phase were also repeated in the correction phase, thus affirming the veracity of those headlines. In the re-evaluation phase (Phase 3), participants read the true and false headlines from the initial evaluation phase again without veracity labels, re-rated the veracity of each headline, and then rated their confidence that they remembered each headline being corrected.

Based on related studies of correction effects on memory and belief updating (Kemp et al., 2022a, 2022b, 2024b; Wahlheim et al., 2020, 2025), we expected that corrections with labeled false and true headlines (i.e., reminder-based corrections) would lead to the greatest reduction in the perceived veracity of false headlines and best promote the recognition of corrections. This type of remembering should also be associated with reductions in false beliefs for all corrections. If the benefits of reminder-based corrections arise partly because misinformation reminders prepare attention for corrective details, then true details in corrections should be fixated earliest for reminder-based corrections. Moreover, if correction recognition depends on attention to true details during encoding, then such recognition should be associated with more and earlier fixations.

## Methods

### Transparency, openness, and data availability

We report how we determined the samples size, all data exclusions, all manipulations, and all measures. The present research complied with the Institutional Review Board at the University of North Carolina at Greensboro (UNCG; Protocol # IRB-FY23-308). The deidentified data, analysis code, and stimulus materials are available on the OSF (https://osf.io/jfvea/).

### Participants

Our stopping rule was to test as many participants as possible over the course of one semester, ending with a final sample that included equal numbers of participants in each of four experimental formats to ensure complete counterbalancing of the stimulus materials (more details appear below). We tested 54 undergraduates from UNCG and compensated them with course credit. We excluded data from one participant whose eyes could not be tracked. We also excluded data from an extra participant, who we tested last, to maintain a balanced assignment of stimuli to conditions. The final sample included 52 participants (39 women, 11 men, 2 non-binary) ages 18–29 years (*M* = 19.27, *SD* = 2.25). The distribution of self-reported racial identity was: 44.23% White, 25% Black or African American, 19.23% Hispanic or Latino, 7.69% Multiple Races, and 3.85% Asian. The years of education for the sample were 12–16 (*M* = 12.94, *SD* = 1.00). The highest level of school or degree for the sample was high school graduate (78.85%) or some college (21.15%). Most participants spoke English as their first language (88.46%), while a minority reported that English was not their first language (11.54%).

### Design

The experiment used a within-subjects design with Headline Type as the independent variable, including four levels. Each participant viewed intermixed lists including false headlines in the initial evaluation phase that were corrected by true headlines without veracity labels in the correction phase (corrections using unlabeled true headlines), false headlines in the initial evaluation phase that were corrected by true headlines with veracity labels in the correction phase (corrections using labeled true headlines), false headlines in the initial evaluation phase that were corrected by false headlines that appeared just before true headlines, both with veracity labels, in the correction phase (corrections using labeled false and true headlines), and true headlines in the initial evaluation phase that repeated in the correction phase without veracity labels (affirmations using true headlines).

### Materials

**Fig. 1:**
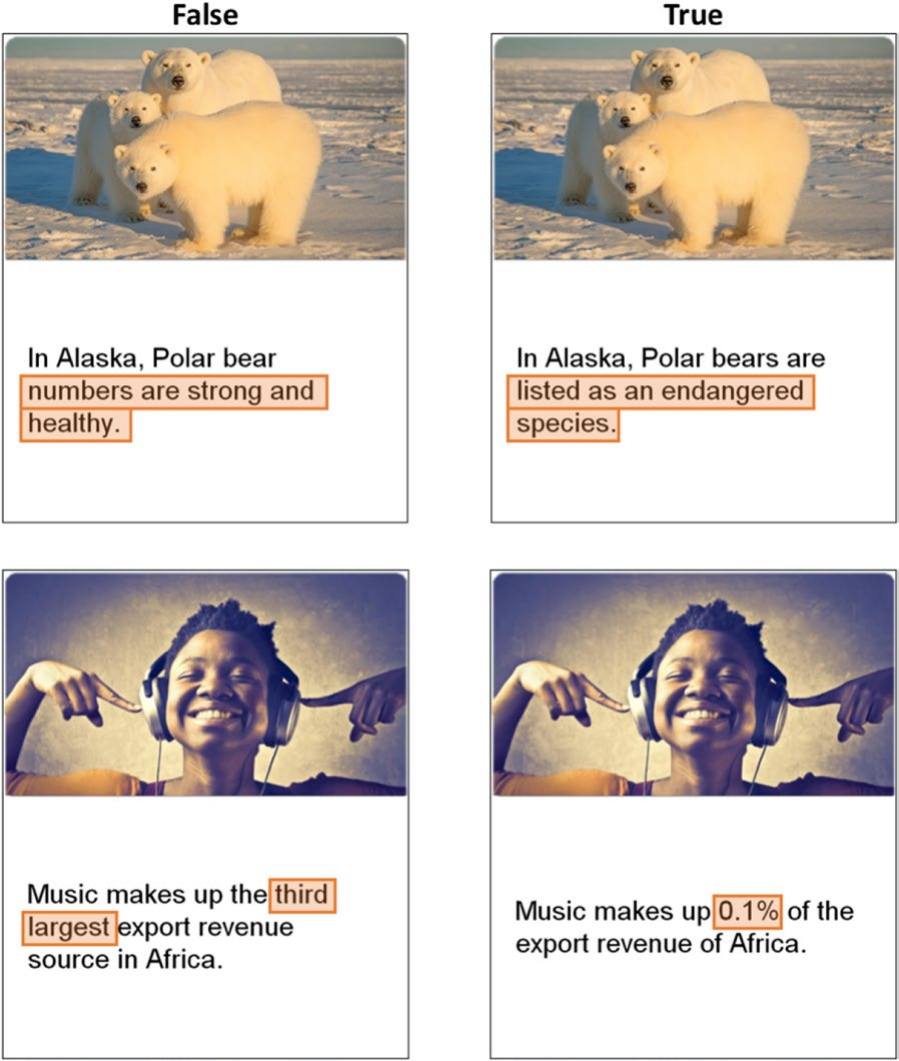
Examples of false and true headline pairs and key details. *Note*: The highlighted boxes above indicate areas of interest surrounding key details and did not appear during the experiment. Key details maintained the same locations for false and true versions for some headline pairs (top row), and slightly changed locations for other headline pairs (bottom row)

The stimuli included 48 critical headline pairs and 24 filler headlines. Each critical pair consisted of a true and false version of the headline and the same topically related image for both versions (see Fig. 1 for example headlines). The filler headlines were always true, appeared without veracity labels, and were included to equate the number of true and false headlines that participants read in each phase. The latter design feature was intended to minimize response biases in perceived veracity ratings (i.e., self-reported beliefs). These filler headlines were also included to encourage consideration of whether headlines that appeared without veracity labels in the correction phase corrected or affirmed headlines from the initial evaluation phase. For counterbalancing, the 48 critical pairs were divided into four groups of 12 and rotated through the within-subjects conditions so that the topics appeared equally often in each condition across four experimental formats.

The headline topics were taken from the fact checking websites Snopes.com, PolitiFact.com, and FactCheck.org. The complete stimulus set is available on the OSF: https://osf.io/jfvea/. Headlines were revised so that all syntax was identical for true and false headlines except the key details that determined the veracity of the headline. The locations of those key details served as the interest areas in the eye tracking analyses (see the highlighted text in Fig. 1 for examples). Although research on misinformation corrections is often concerned with political issues, especially because partisanship can influence how news is evaluated (e.g., Moravec et al., 2019), we decided not to include highly partisan headline topics to better focus the experiment on the attentional and mnemonic mechanisms of interest. That is, we included less political headline topics to preemptively control for potential interactions between individual differences in participants’ political partisanship and the political leanings of the headline topics as a source of variance in our dependent measures. The legitimacy of this approach is supported by research suggesting that politically neutral topics are valid for assessing misinformation susceptibility and can be used with or in place of partisan topics (Maertens et al., 2024). Of course, topics can become politicized despite appearing neutral. This concern motivated our decision to counterbalance headline topics across conditions; having topics appear at least once in each condition should equalize political thinking evoked by headline topics across experimental formats. We also controlled for variance introduced by individual topics in our mixed-effects model structures, which we report in our Statistical Methods section below.

To assess fixations on key headline details—which are details that could be false in the initial evaluation phase and subsequently replaced with true details during the correction phase—we drew parallelograms around these details (see example areas of interest in Fig. 1) and recorded counts and associated timing in those areas of interest over the 8 s presentation intervals. Note that these parallelograms were not visible to participants; all headline text appeared without any indication of the areas of interest. We summed the counts across separate parallelograms when multiple areas of interest were required to cover the entirety of the key details, such as in the case of line breaks (Fig. 1, top). This approach ensured that areas of interest encompassed all key details while excluding other text and whitespace.

### Procedure

All participants were tested individually, seated at a computer, with either one or two experimenter(s) present. Stimuli appeared on a 24 in. monitor (1920 × 1080 pixel resolution) at a 16:9 pixel aspect ratio and their presentation was controlled by Experiment Builder software (version 2.4.1; SR Research, Mississauga, Ontario, Canada).

Participants began the session by answering eight questions about their demographics (for a complete list of questions, see the OSF: https://osf.io/jfvea/). After the demographic questions, participants placed their heads against a chin and forehead rest to minimize motion. Gaze location was recorded from the right eye using an infrared pupil–corneal eye tracker (EyeLink 1000 Plus; SR Research, Mississauga, Ontario, Canada) that sampled at 1000 Hz. The camera was positioned 55 cm from the top of the rest. The viewing distance was 58 cm from the rest, and the viewing angle was 32°/25°. Before starting the experiment, the eye tracker was calibrated and validated. Calibration entailed participants fixating on targets in a 3 × 3 array. Calibration accuracy was confirmed using a validation procedure during which participants fixated on a target that moved to different screen positions. In all phases, a cross appeared in the center of the screen at the beginning of each trial. A drift correction was triggered when participants fixated on the cross for less than 0.5 s. The drift correction required participants to fixate on the cross until an experimenter confirmed the fixation. The trial would not proceed past the cross until the participant’s gaze was detected on the cross for 0.5 s within 4 s of it appearing.

**Fig. 2:**
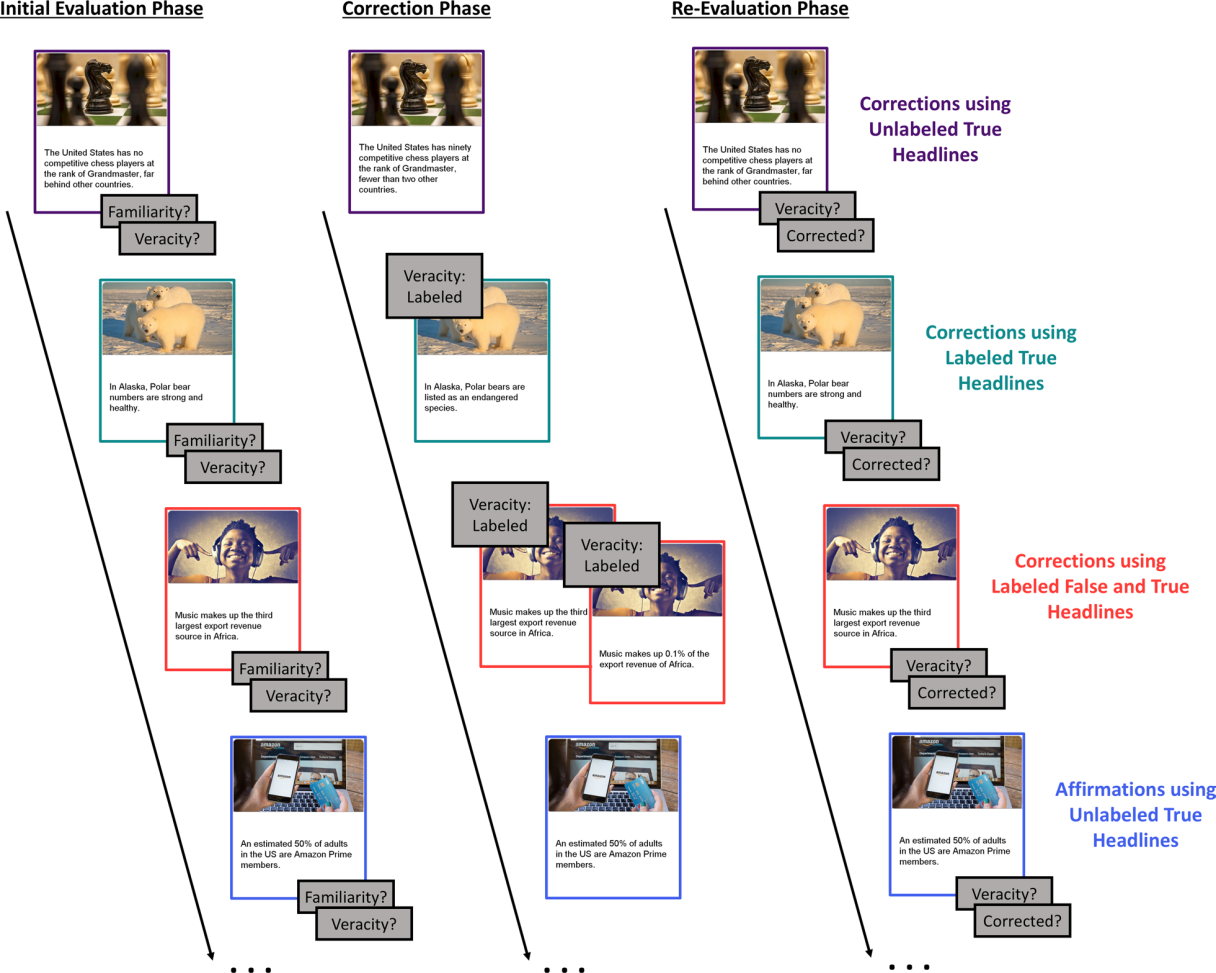
Schematic of the procedure. *Note:* In the initial evaluation phase, participants saw a headline of unclear veracity for 8 s and had unlimited time to rate headline familiarity and veracity. In the correction phase, headlines appeared as follows: Corrections using unlabeled true headlines (purple) and affirmations using unlabeled true headlines (blue) appeared for 8 s; Corrections using labeled true headlines (green) appeared for 8 s after a veracity label that appeared for 3 s; Corrections using labeled false and true headlines (red) displayed a reminder label for 3 s, a false reminder headline for 8 s, a veracity label for 3 s, and a true headline for 8 s. In the re-evaluation phase, participants saw a headline of unclear veracity for 8 s and had unlimited time to rate headline veracity and confidence that the headline was corrected in the correction phase

Figure 2 displays a schematic of the experimental procedure. In the initial evaluation phase, participants were instructed to read true and false headlines and to make ratings about their preexisting familiarity with and the veracity of each headline. Before each trial, a fixation cross appeared for 4 s followed by a 1 s interstimulus interval (ISI) unless a drift correction was triggered. A headline then appeared for 8 s followed by a prompt to rate its familiarity on a scale of 1 (not at all familiar) to 4 (very familiar). Participants made ratings by pressing corresponding buttons on a button box indicated by an on-screen diagram. After a response was registered, a prompt to rate the veracity of the headline appeared. Participants rated veracity on a scale of 1 (not at all accurate) to 4 (very accurate) using the same buttons as before, indicated by a diagram on the screen. One version of each headline appeared for 72 total trials in a random order.

In the correction phase, participants were told that they would read true headlines on the same topics from the initial evaluation phase. They were told that true headlines would be affirmed and that false headlines would be corrected using three different formats. Participants were given an example of how each correction format would appear. Before each trial, a fixation cross appeared for 4 s followed by a 1 s ISI unless a drift correction was triggered. For filler headlines as well as headlines in the corrections and affirmations using unlabeled true headlines conditions, a true headline appeared for 8 s without any indication of its veracity. For corrections using labeled true headlines, a label indicating that the following headline would correct false information from the initial evaluation phase appeared for 3 s. Then the true headline appeared for 8 s. For corrections using labeled true and false headlines, a label indicating that the following headline would show false information from the initial evaluation phase appeared for 3 s. Then the false headline seen in the initial evaluation phase appeared for 8 s. Next, a label indicating that the following headline would correct false information from the initial evaluation phase appeared for 3 s. Then the true version of the same headline appeared for 8 s. Participants saw a total of 12 headlines in each critical condition and 24 filler headlines, which appeared in a random order. Each headline topic was shown once for a total of 72 trials.

The re-evaluation phase followed the same structure as the initial evaluation phase. Participants were told that they would read the same headlines that appeared in the initial evaluation phase. They were instructed to rate the veracity of each headline and their confidence in remembering that each headline had been corrected in the correction phase. The fixation cross appeared for 4 s followed by a 1 s ISI unless a drift correction was triggered. A headline without veracity information appeared for 8 s followed by a prompt to rate the veracity of the headline on a scale of 1 (not at all accurate) to 4 (very accurate). Participants made ratings by selecting the corresponding button on the button box indicated by an on-screen diagram. A prompt to rate their memory for whether the headline was corrected in the correction phase appeared next. Participants made ratings on a scale of 1 (not corrected) to 4 (definitely corrected) using the buttons indicated by an on-screen diagram. One version of each headline appeared for a total of 72 trials.

### Statistical methods

We used R software (R Core Team, 2023) to conduct all analyses and examined the effects of interest using mixed-effects models from the lme4 package (Bates et al., 2015). Four sets of models corresponded to different behavioral outcomes. These outcomes included familiarity, veracity ratings, high confidence recognition of corrections, and veracity ratings conditioned on high confidence recognition of corrections. Familiarity and veracity ratings were measured on 1–4 rating scales and were fitted with linear mixed-effects models (function lmer). We binarized the ratings of correction recognition such that high confidence recognition was indicated by the 4 responses, while responses 1–3 were combined into the other category. We excluded the second highest rating (3, probably corrected) from this operational definition of correction recognition because the lack of complete certainty implied that recollection of contextual information contributed less to these judgments. Of course, we cannot ensure, and do not claim, that this memory measure is process pure. We modeled these binary responses using logistic mixed-effects models (function glmer).

Six sets of models corresponded to different eye tracking outcomes. The number of fixations was the primary outcome, but we also measured the time associated with fixations. Specifically, the outcomes included fixation counts, time to first fixation, fixation counts conditioned on time bin, fixation counts conditioned on correction recognition, time to first fixation conditioned on correction recognition, and fixation counts conditioned on time bin and correction recognition. These variables were measured continuously and were fitted with linear mixed-effects models (function lmer). Because we were primarily interested in how correction format influenced fixations to key headline details, we only included the three correction conditions as levels of the headline type fixed effect in the eye tracking analyses (i.e., we omitted the affirmations condition). However, we did analyze the contributions of all headline types, including affirmations, in these effects using a separate set of models with identical specifications to those described below. The observed significant differences in estimated marginal means among the correction headline type conditions were comparable regardless of whether the affirmation condition was included in the models.

All models for behavioral and eye tracking analyses included by-subject and by-item random intercept effects. We performed Wald’s Chi-square hypothesis tests using the ANOVA function of the car package (Fox & Weisberg, 2019) and pairwise comparisons correcting for multiple comparisons using the Tukey method in the emmeans package (Lenth, 2021). We report the observed power (*OP*) to detect observed effect sizes for each model based on the results of a sensitivity analysis conducted with the powerSim function from the simR package (Green & MacLeod, 2016). Because our model specifications included categorical variables with multiple levels as fixed effects, we used a likelihood ratio test to estimate *OP* for a significant effect across all levels of the fixed effect unless otherwise noted. We estimated *OP* to detect the highest order interaction in each model specification since these interactions require more power to detect than lower-order interactions and main effects. We also estimated *OP* to detect significant lower-order interactions or main effects when the highest order interactions were not significant. Effect sizes for all models were calculated using the r2 function from the partR2 package (Stoffel et al., 2024). These effect sizes indicate the proportion of variance explained by the fixed and random effects (conditional *R*^2^ [*R*_*c*_^2^]) and fixed effects alone (marginal *R*^2^ [*R*_*m*_^2^]). We calculated effect sizes for pairwise comparisons using Cohen’s *d* for linear mixed-effects models and odds ratios (*OR*) for logistic mixed-effects models using the eff_size and emmeans functions from the emmeans package (Lenth, 2021). All model specifications are available in the analysis scripts on the OSF: https://osf.io/jfvea/. The significance level was *α* = 0.05.

The eye movement data were processed using Data Viewer software (version 4.3.2; SR Research, Mississauga, Ontario, Canada). Fixations were identified and assigned to interest areas by the online parsing system (EyeLink 1000 Plus; SR Research, Mississauga, Ontario, Canada). The position of a fixation was determined by the average position of all samples that made up the fixation. The onset of a fixation was determined by the offset of the previous saccade while the offset of a fixation was determined by the onset of the following saccade. Saccades were detected by the parser by comparing eye movements to motion, velocity, and acceleration thresholds. Movements of more than 0.15° with a velocity and acceleration exceeding 30°/s and 8000°/s^2^, respectively, were classified as saccades. Fixations were assigned to 1 s bins within the 8 s headline viewing period. Downsampling was not used in the analyses.

## Results

A minority of headlines (three topics) contained statements that were corrected with direct opposite statements (e.g., “There is no law requiring the President to turn over tax documents to Congress” was corrected by “There is a law requiring the President to turn over tax documents to Congress”). Participants may have implicitly corrected these headlines with their opposite statements when they appeared following false veracity labels, potentially amplifying corrective effects of reminders observed in the re-evaluation phase. Including headline topic as a random effect should have minimized variance introduced by these topics. As an additional precaution, we reran the analyses with these headlines removed and verified the same patterns of results reported here. Those model specifications and results are available on the OSF (https://osf.io/jfvea/).

### Behavioral self-report measures

#### Initial evaluation phase: familiarity ratings

**Fig. 3:**
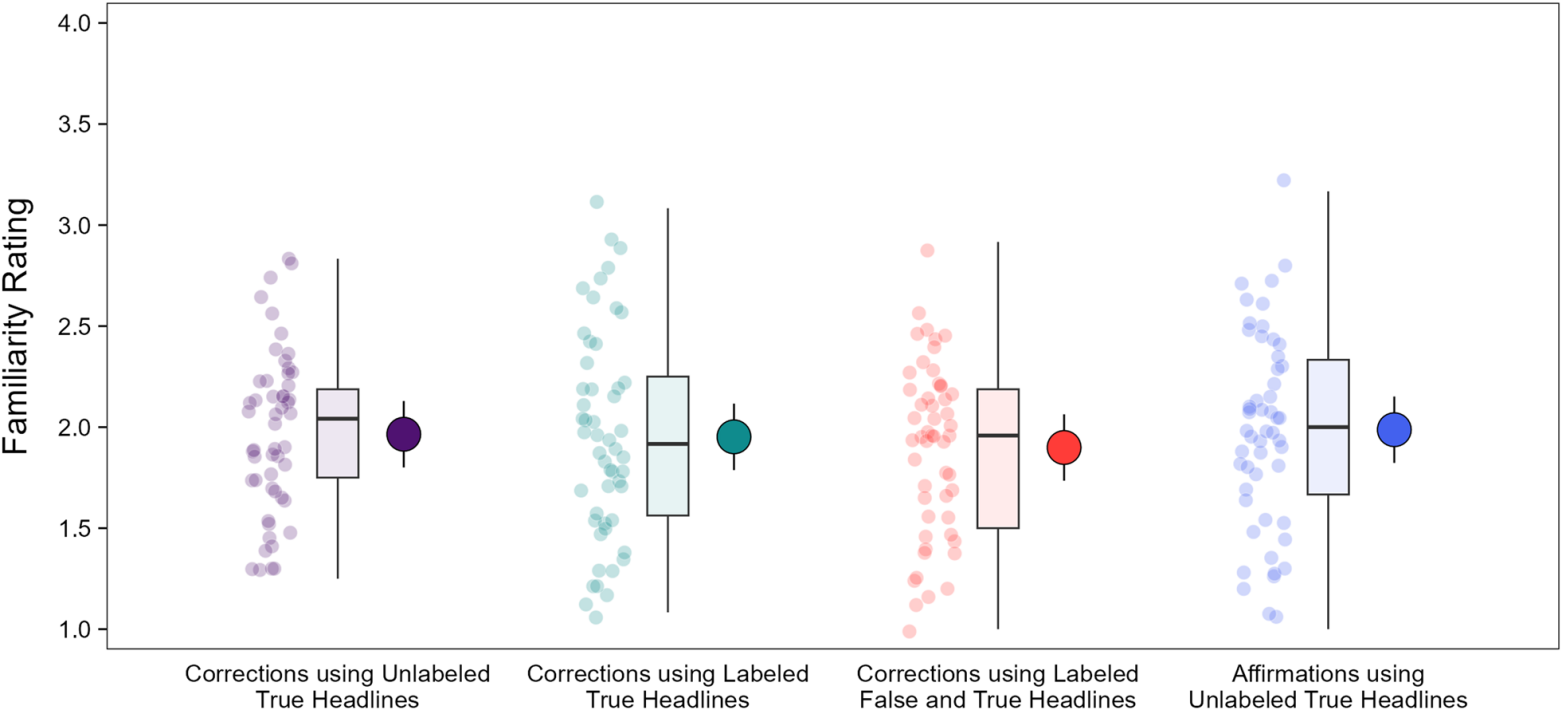
Familiarity ratings in the initial evaluation phase. *Note*: For each condition, the leftmost points are individual participant means. The boxplots show the interquartile ranges, medians, and variability 1.5 times the interquartile range. The rightmost points are model population estimates with 95% confidence intervals (error bars)

To confirm that the counterbalancing resulted in headline topics being comparably familiar across conditions, we compared familiarity in the initial evaluation phase across headline types (Fig. 3). A model with headline type as a fixed effect [*R*_*m*_^2^ = 0.00, *R*_*c*_^2^ = 0.32] indicated no significant effect, χ^2^(3) = 4.19, *p* = 0.242, *OP* = 35%. This showed that familiarity was comparable across headline types in the initial evaluation phase. Moreover, the estimates indicated that, on average, the headlines were relatively low in familiarity.

#### Initial evaluation phase: veracity ratings

**Fig. 4:**
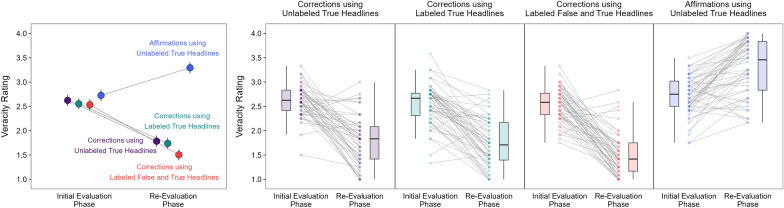
Perceived veracity ratings in the initial evaluation and re-evaluation phases. *Note*: The left panel shows perceived veracity ratings for all headline types. The points are model population estimates with 95% confidence intervals (error bars). Participants rated the veracity of false headlines in the correction conditions (purple, green, and red points) and true headlines in the affirmation condition (blue points). The right panel shows perceived veracity ratings for individual participants in the correction conditions (first three panels) and the affirmation condition (rightmost panel). The boxplots show the interquartile ranges, medians, and variability 1.5 times the interquartile range. The points are individual participant means. Variation in point opacity reflects differences in the number of participants (i.e., darker points = more participants). The line slopes show rating change magnitudes across participants

To determine how corrections and affirmations affected veracity ratings, we examined the changes in veracity ratings from the initial evaluation phase to the re-evaluation phase. Figure 4 displays these changes in aggregate (left panel) and for individual participants (right panels). A model with phase and headline type as fixed effects [*R*_*m*_^2^ = 0.27, *R*_*c*_^2^ = 0.36] showed significant effects of phase, χ^2^(1) = 447.33, *p* < 0.001, and headline type, χ^2^(3) = 974.88, *p* < 0.001, and a significant interaction, χ^2^(3) = 654.01, *p* < 0.001, *OP* = 100%.

Baseline ratings in the initial evaluation phase did not differ across false headlines in the correction conditions, largest z ratio = 1.80, *p* = 0.275, *d* = 0.10, and did not differ between true headlines (blue point) and false headlines that were eventually corrected with unlabeled true headlines (purple point), z ratio = 2.05, *p* = 0.169, *d* = 0.12. However, baseline ratings (blue point) were significantly higher for true headlines than false headlines in the remaining two correction conditions (green and red points), smallest z ratio = 3.46, *p* = 0.003, *d* = 0.20. Therefore, with only one exception, participants perceived false headlines to be, on average, less accurate than true headlines.

From the initial evaluation phase to the re-evaluation phase, veracity ratings decreased significantly for corrected false headlines and increased significantly for affirmed true headlines, smallest z ratio = 11.39, *p* < 0.001, *d* = 0.65. In the re-evaluation phase, the comparison of false headlines in the correction conditions showed significantly lower ratings for corrections using labeled false and true headlines (red point) than for the other two correction types (purple and green points), smallest z ratio = 4.68, *p* < 0.001, *d* = 0.27. Ratings were not significantly different between the other correction conditions (i.e., those using unlabeled or labeled true headlines only), z ratio = 0.90, *p* = 0.806, *d* = 0.05. These results replicate earlier findings showing that reminder-based corrections led to the lowest false beliefs on an immediate test (Wahlheim et al., 2025).

#### Re-evaluation phase: correction recognition

**Fig. 5:**
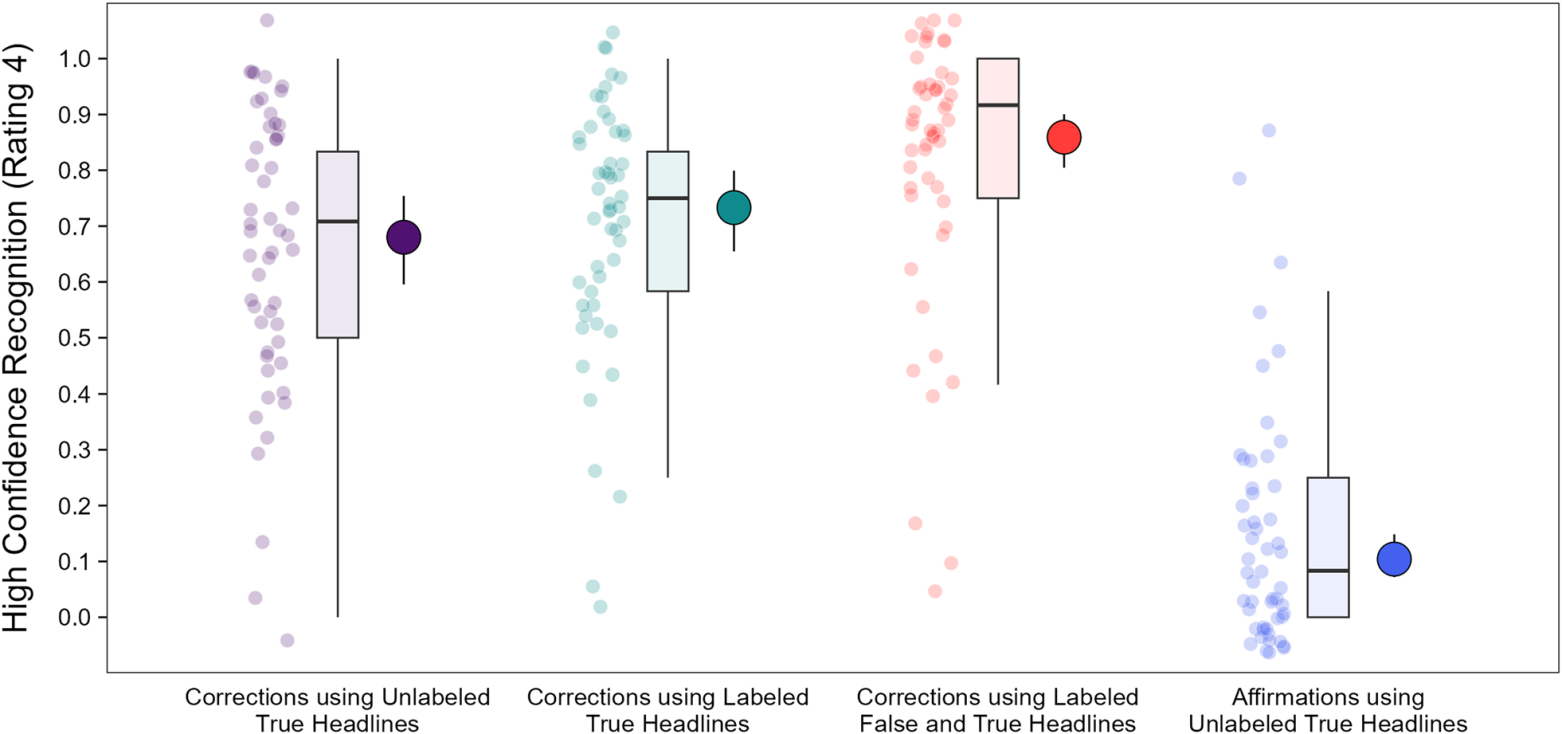
Probabilities of high confidence recognition of corrections in the re-evaluation phase. *Note*: Probabilities of participants giving the highest rating (4, definitely corrected) when indicating their memory for corrections (i.e., correction recognition). The leftmost points are individual participant means. The boxplots show the interquartile ranges, medians, and variability 1.5 times the interquartile range. The rightmost points are model population estimates with 95% confidence intervals (error bars)

Figure 5 displays the probabilities of correction recognition, which was operationally defined as the highest rating that participants could give when indicating their memory for whether headlines were corrected (4, definitely corrected). The highest memory rating (4) was accurate for the correction conditions (purple, green, and red points) and inaccurate for the affirmation condition (blue point). A model with headline type as a fixed effect [*R*_*m*_^2^ = 0.33, *R*_*c*_^2^ = 0.52] indicated a significant effect, χ^2^(3) = 516.19, *p* < 0.001, *OP* = 100%. The comparison of correction conditions showed significantly higher correction recognition for corrections using labeled false and true headlines (red point) than the other correction types (purple and green points), smallest z ratio = 5.31, *p* < 0.001, *OR* = 2.22. Correction recognition did not significantly differ between the other correction types (i.e., those using unlabeled or labeled true headlines only), z ratio = 1.90, *p* = 0.227, *OR* = 1.29. Finally, accurate correction recognition ratings in the correction conditions were significantly higher than the incorrect ratings in the affirmation condition, smallest z ratio = 17.79, *p* < 0.001, *OR* = 18.28. Collectively, these results show that misinformation reminders best promoted high confidence recognition of corrections, again replicating prior work (Wahlheim et al., 2025).

#### Initial evaluation and re-evaluation phases: veracity ratings conditioned on correction recognition

**Fig. 6:**
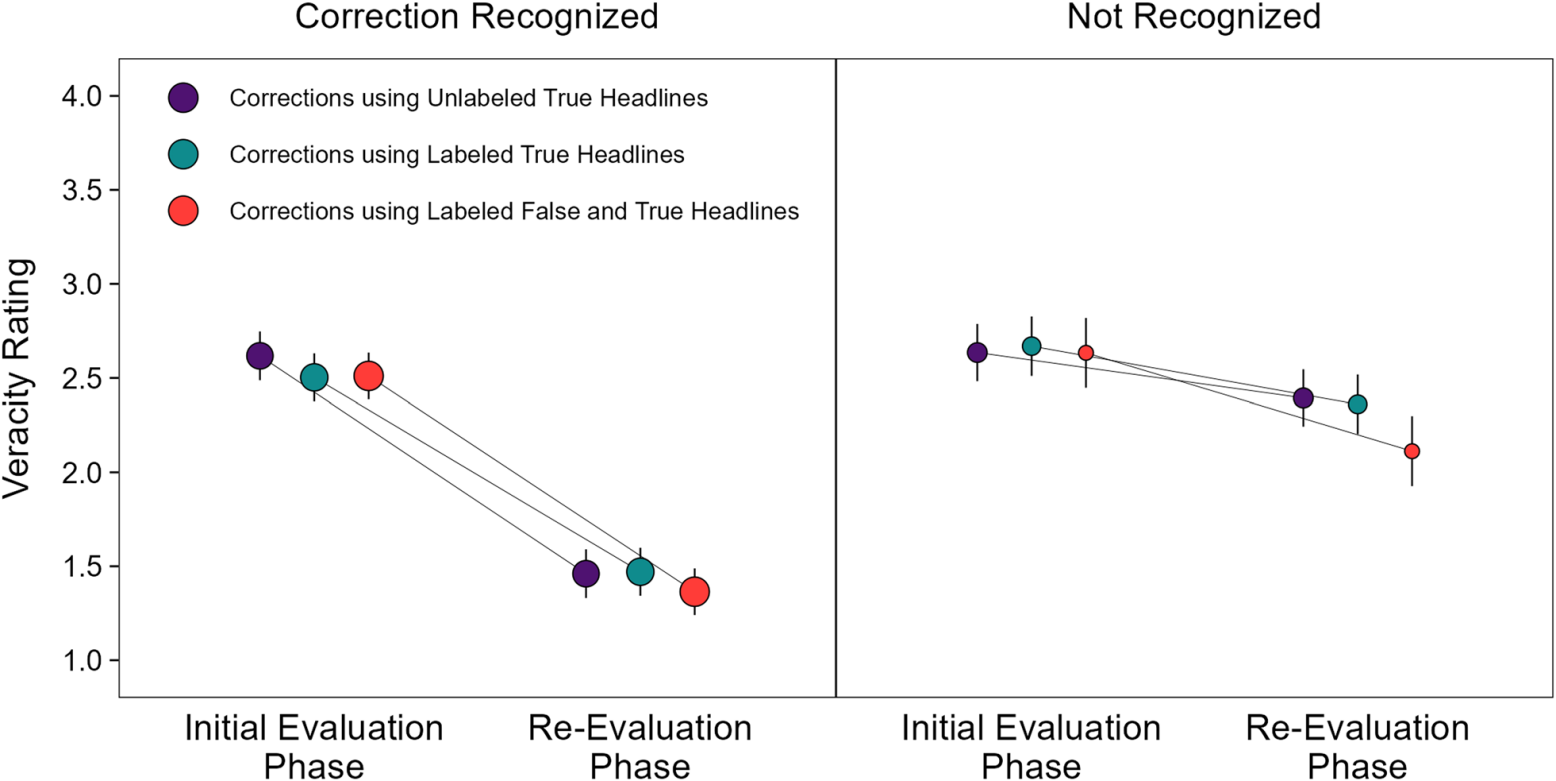
Perceived veracity ratings in the initial evaluation and re-evaluation phases conditioned on correction recognition in the re-evaluation phase. *Note*: Perceived veracity ratings for the correction conditions when corrections were recognized (left panel) and not recognized (right panel). High confidence recognition reflected memory for corrections with the highest rating (4), and unrecognized corrections reflected memory for corrections collapsed across all other ratings (1–3). The points are model population estimates with 95% confidence intervals (error bars). Variations in point areas indicate relative differences in the number of observations across conditional cells

Figure 6 displays changes in veracity ratings from the initial evaluation phase to the re-evaluation phase when corrections were recognized (left panel) and not recognized (right panel) in the correction conditions. Table 1 displays the results from a mixed-effects model with fixed effects of phase, headline type, and correction recognition [*R*_*m*_^2^ = 0.26, *R*_*c*_^2^ = 0.37]. We do not elaborate on main effects redundant with those reported above. The model indicated significant effects of headline type, phase, and correction recognition, smallest χ^2^(2) = 10.40, *p* = 0.006, and a significant phase by correction recognition interaction, χ^2^(1) = 157.52, *p* < 0.001, *OP* = 100%. No other effects were significant, largest χ^2^(2) = 3.82, *p* = 0.148, *OP* = 40%. The interaction showed that the significant reduction in the perceived veracity of false headlines across phases was greater when corrections were recognized, z ratio = 34.50, *p* < 0.001, *d* = 1.33, than when corrections were not recognized, z ratio = 6.68, *p* < 0.001, *d* = 0.43. Thus, these results show strong associations between correction recognition and reductions in the perceived veracity of false headlines, which replicates prior findings (Wahlheim et al., 2025).

**Table 1:** Model results for veracity ratings across correction headlines and phases conditioned on correction recognition

Effect	χ^2^	*df*	*p*
Headline type	10.40	2	= .006
Phase	1076.27	1	< .001
Correction recognition	180.41	1	< .001
Headline type × phase	3.57	2	= .167
Headline type × correction recognition	1.34	2	= .511
Phase × correction recognition	157.52	1	< .001
Headline type × phase × correction recognition	3.82	2	= .148

## Summary of behavioral self-report measures

The patterns of perceived veracity and correction recognition confirmed that, as shown more generally in the misinformation correction literature, reminder-based corrections were most effective in reducing the perceived veracity of false headlines and promoting subsequent memory for corrections. The association between the recognition of corrections and false belief reduction suggests that perceptions of headline veracity were based partly on how well participants remembered that the corrections occurred. If such remembering depends on attention during encoding of true details, then the overall benefits of reminder-based corrections may reflect more effective allocation of attention to corrective information. We examine this possibility next.

### Eye tracking measures of attention during encoding

#### Fixation counts in all phases

**Fig. 7:**
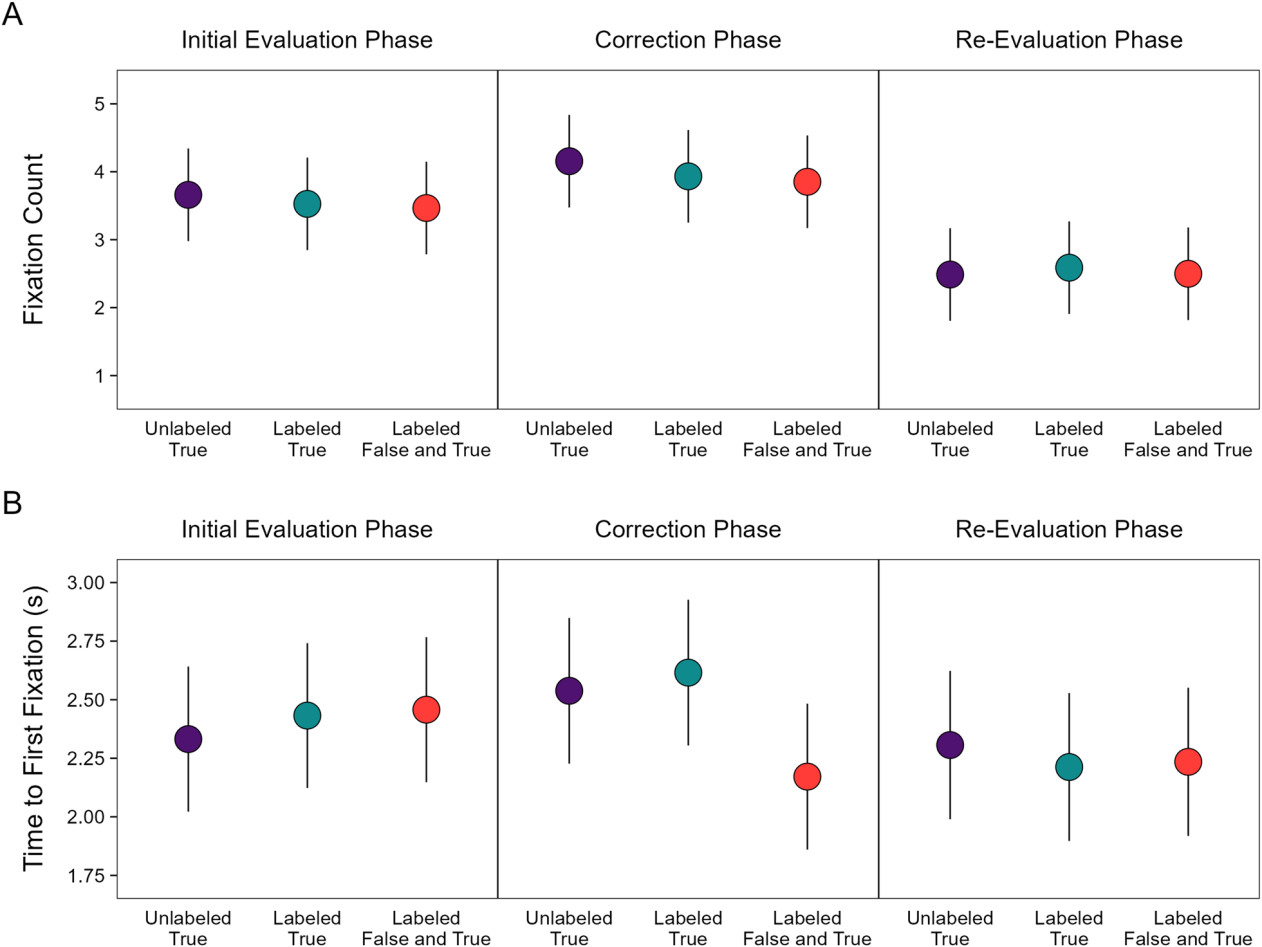
Fixation counts and time to first fixation for correction headline types. *Note*: Number of fixations on key details (**A**) and time to first fixation on a key detail (**B**). The points are model population estimates with 95% confidence intervals (error bars)

We first characterized the overall number of fixations to key details across all phases (Fig. 7A). A model with headline type and phase as fixed effects [*R*_*m*_^2^ = 0.03, *R*_*c*_^2^ = 0.44] indicated a significant effect of phase, χ^2^(2) = 281.42, *p* < 0.001, *OP* = 100%, no significant effect of headline type, χ^2^(2) = 3.30, *p* = 0.192, and no significant interaction, χ^2^(4) = 2.99, *p* = 0.559, *OP* = 27%. Fixation counts were significantly different across all phases, smallest z ratio = 4.81, *p* < 0.001, *d* = 0.16, in the following order: correction phase > initial evaluation phase > re-evaluation phase. These results suggest that participants fixated more on corrective details than the original key details, possibly because noticing the changes in the correction phase held their attention longer. The re-evaluation phase showing the lowest counts may reflect participants disengaging after reaching their conclusions about the veracity of headlines and whether they were corrected.

#### Time to first fixation in all phases

To further characterize fixation patterns across phases, we modeled the time to first fixation on key details [*R*_*m*_^2^ = 0.01, *R*_*c*_^2^ = 0.36] (Fig. 7B). The model indicated significant effects of headline type, χ^2^(2) = 7.46, *p* = 0.024, and phase, χ^2^(2) = 14.38, *p* < 0.001, as well as a significant interaction, χ^2^(4) = 25.04, *p* < 0.001, *OP* = 100%. The interaction showed that there were no significant differences in the time to fixate key details across correction conditions in the initial evaluation and re-evaluation phases, largest z ratio = 1.48, *p* = 0.301, *d* = 0.09, but in the correction phase, key details were fixated significantly faster for corrections using labeled false and true headlines (red point) than the other correction types that did not include reminders of false information (purple and green points), smallest z ratio = 4.17, *p* < 0.001, *d* = 0.27. Collectively, these results suggest that misinformation reminders directed attention to subsequent true details and initiated encoding of that corrective information earlier than in the other correction conditions.

#### Fixation counts over time in the correction phase

**Fig. 8:**
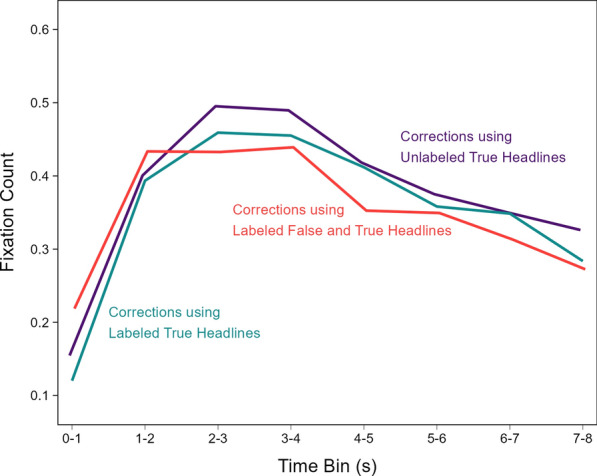
Fixations on key details in the correction phase over time. *Note*: The lines connect model population estimates at each time bin

The differences in veracity ratings, correction recognition, and time to first fixation above suggest that key true details were encoded differently across conditions and that this difference may have manifested as differences in the temporal dynamics of attention allocation in the correction phase. To examine this possibility further, we decomposed the aggregate correction phase fixation counts into time courses comprising eight 1-s bins (Fig. 8). A model with headline type and time bin as fixed effects [*R*_*m*_^2^ = 0.02, *R*_*c*_^2^ = 0.16] indicated significant effects of headline type, χ^2^(2) = 8.33, *p* = 0.016, and time bin, χ^2^(7) = 563.22, *p* < 0.001, as well as a significant interaction, χ^2^(14) = 32.49, *p* = 0.003, *OP* = 99%.

The interaction indicated that the time bin effect showing the fewest fixations to key details in the first second, followed by a sharp increase, then a gradual decrease until stimulus offset varied across the headline type conditions. The 0–1 s bin included significantly more fixations for corrections using labeled false and true headlines (red line) than for the other correction types (purple and green lines), smallest z ratio = 2.39, *p* = 0.044, *d* = 0.10. Fixations in that bin were not significantly different between the other headline types (i.e., corrections using unlabeled or labeled true information only), z ratio = 1.29, *p* = 0.403, *d* = 0.05. Additionally, the 4–5 s bin included significantly fewer fixations for corrections using labeled false and true headlines (red line) than corrections using unlabeled true headlines (purple line), z ratio = 2.45, *p* = 0.038, *d* = 0.10. Fixations in that bin did not significantly differ among the other conditions, largest z ratio = 2.21, *p* = 0.069, *d* = 0.09. No other comparisons revealed significant differences, largest z ratio = 2.33, *p* = 0.051, *d* = 0.09. These results show that repeating false information during corrections led to more fixations at the start of viewing the correct headline, and slightly fewer fixations in middle–later viewing periods. This pattern is consistent with the time to first fixation findings in suggesting that misinformation reminders directed participants’ initial attention to key details earlier, which provided more time for elaborative encoding, and/or allowed participants to complete their encoding earlier.

#### Fixation counts in the correction phase conditioned on correction recognition in the re-evaluation phase

**Fig. 9:**
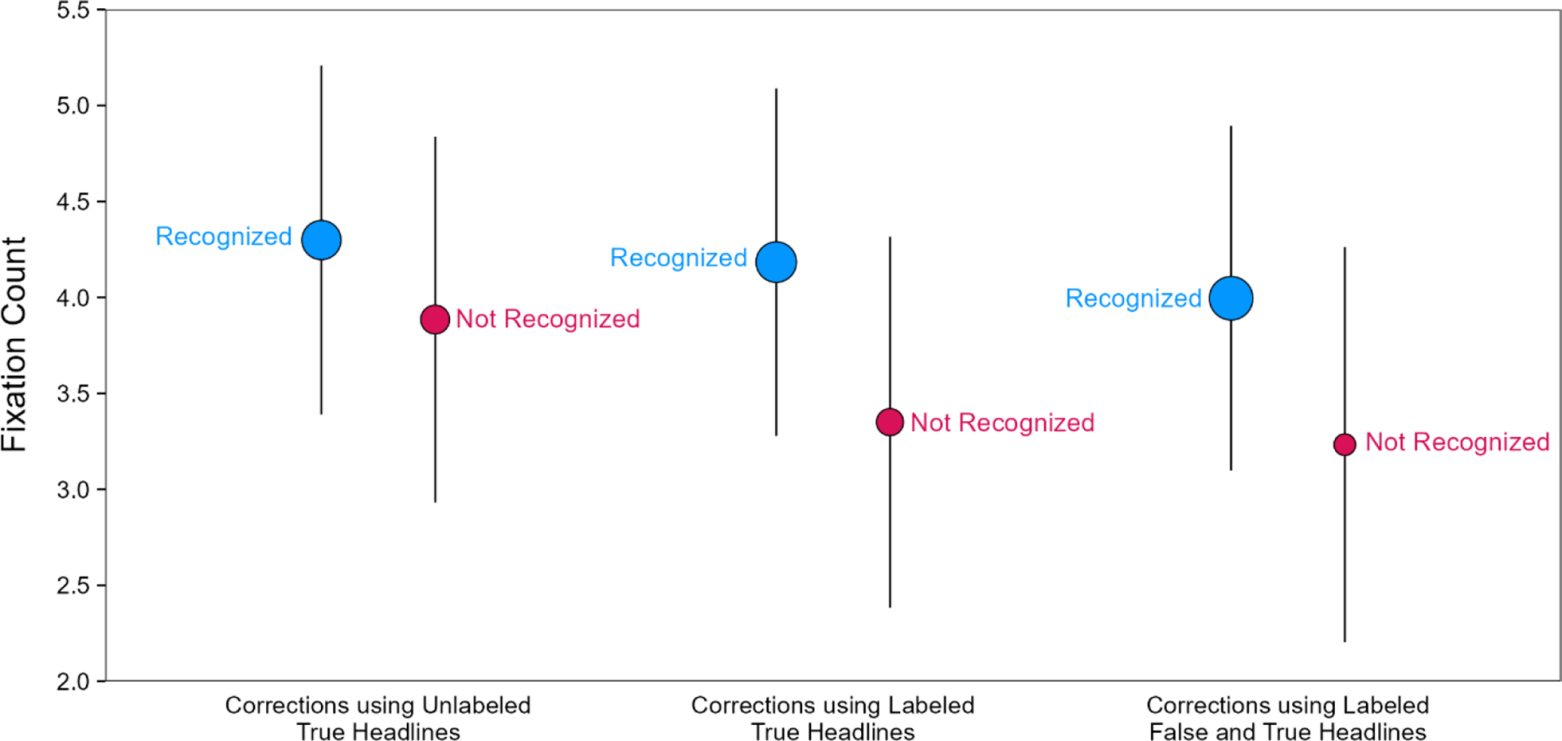
Fixations on key details in the correction phase conditioned on correction recognition in the re-evaluation phase. *Note*: The points are model population estimates with 95% confidence intervals (error bars). Variation in point size signals differences in the number of observations

The differences in fixation timing across the correction conditions in the correction phase—taken with the differences in correction recognition in the re-evaluation phase—suggest that trial-to-trial fixation differences may have determined whether corrections were subsequently recognized, similar to findings showing that more fixations to key objects was associated with remembering changes (Wahlheim et al., 2022). This leads to the possibility that recognized corrections received more fixations during the correction phase, which increased attention to the conflicting false and true details. We examined this possibility using a subsequent memory analysis in which we modeled correction phase fixations conditioned on whether corrections were recognized in the re-evaluation phase (Fig. 9). A model with headline type and correction recognition as fixed effects [*R*_*m*_^2^ = 0.01, *R*_*c*_^2^ = 0.52] indicated no significant effect of headline type, χ^2^(2) = 5.93, *p* = 0.052, a significant effect of correction recognition, χ^2^(1) = 12.71, *p* < 0.001, *OP* = 93%, and no significant interaction, χ^2^(2) = 1.48, *p* = 0.477, *OP* = 15%. Fixation counts were significantly higher for recognized than unrecognized corrections. Taken with corrections being recognized most for corrections using labeled false and true headlines, these results suggest that reminders promoted the encoding of corrections as such, which supported subsequent memory for veracity information.

**Fig. 10:**
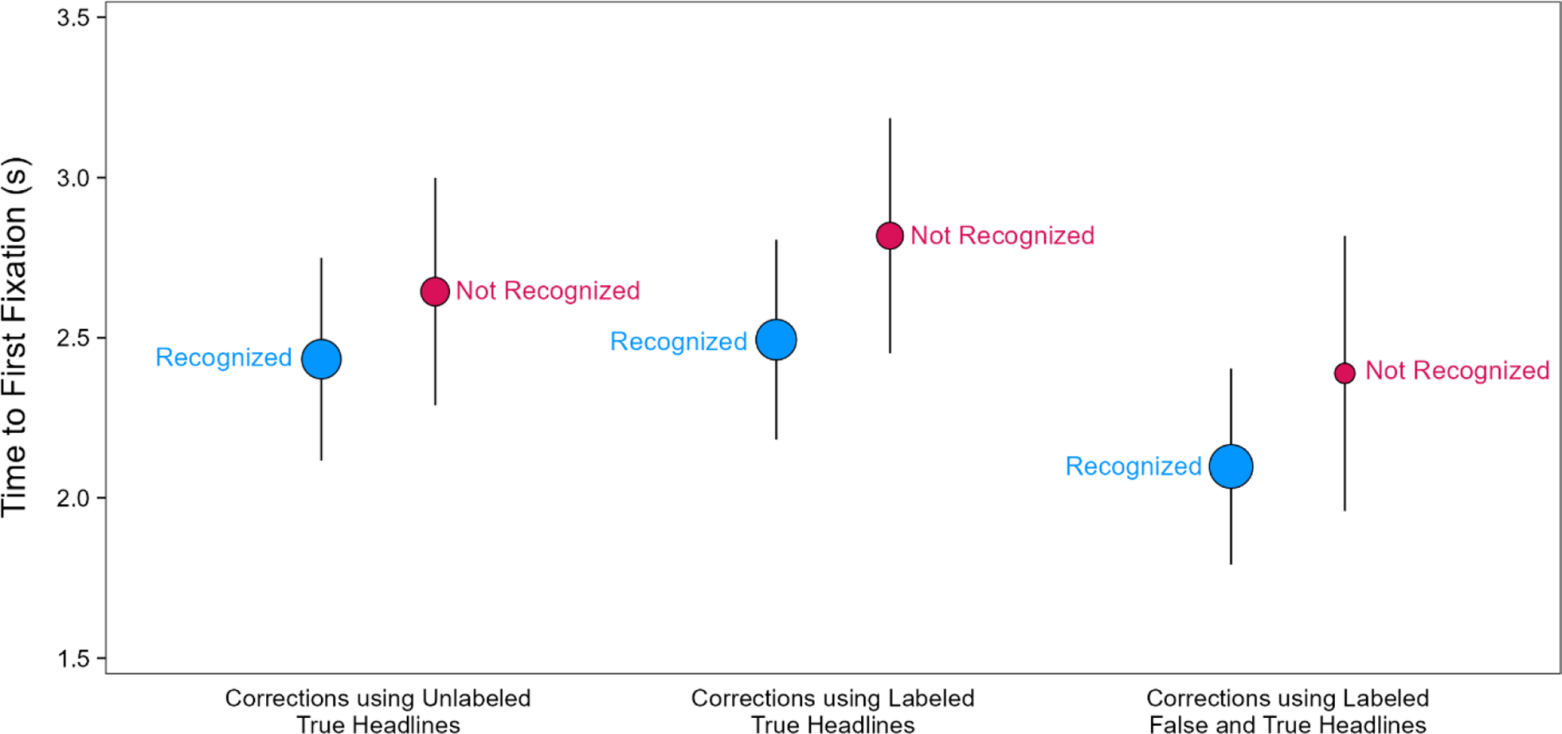
Time to first fixation on key details in the correction phase conditioned on correction recognition over time. *Note*: The points are model population estimates with 95% confidence intervals (error bars). Variation in point size signals differences in the number of observations

Using this subsequent memory approach, we next modeled the time to first fixation in the correction phase conditioned on correction recognition in the re-evaluation phase [*R*_*m*_^2^ = 0.02, *R*_*c*_^2^ = 0.31] (Fig. 10). If attention during encoding of key details in the correction phase promotes correction recognition, then participants may show earlier fixations to the true details of corrections that are subsequently recognized. The model indicated significant effects of headline type, χ^2^(2) = 20.17, *p* < 0.001, *OP* = 98%, and correction recognition, χ^2^(1) = 6.98, *p* = 0.008, *OP* = 74%, and no interaction, χ^2^(2) = 0.32, *p* = 0.853, *OP* = 1%. The effect of correction recognition showed that the average first fixation times were faster for corrections that were subsequently recognized. Taken with the finding that reminder-based corrections led to the best recognition of corrections, these results suggest that misinformation reminders promoted earlier noticing of true details that supported subsequent memory as compared to the other correction formats.

**Fig. 11:**
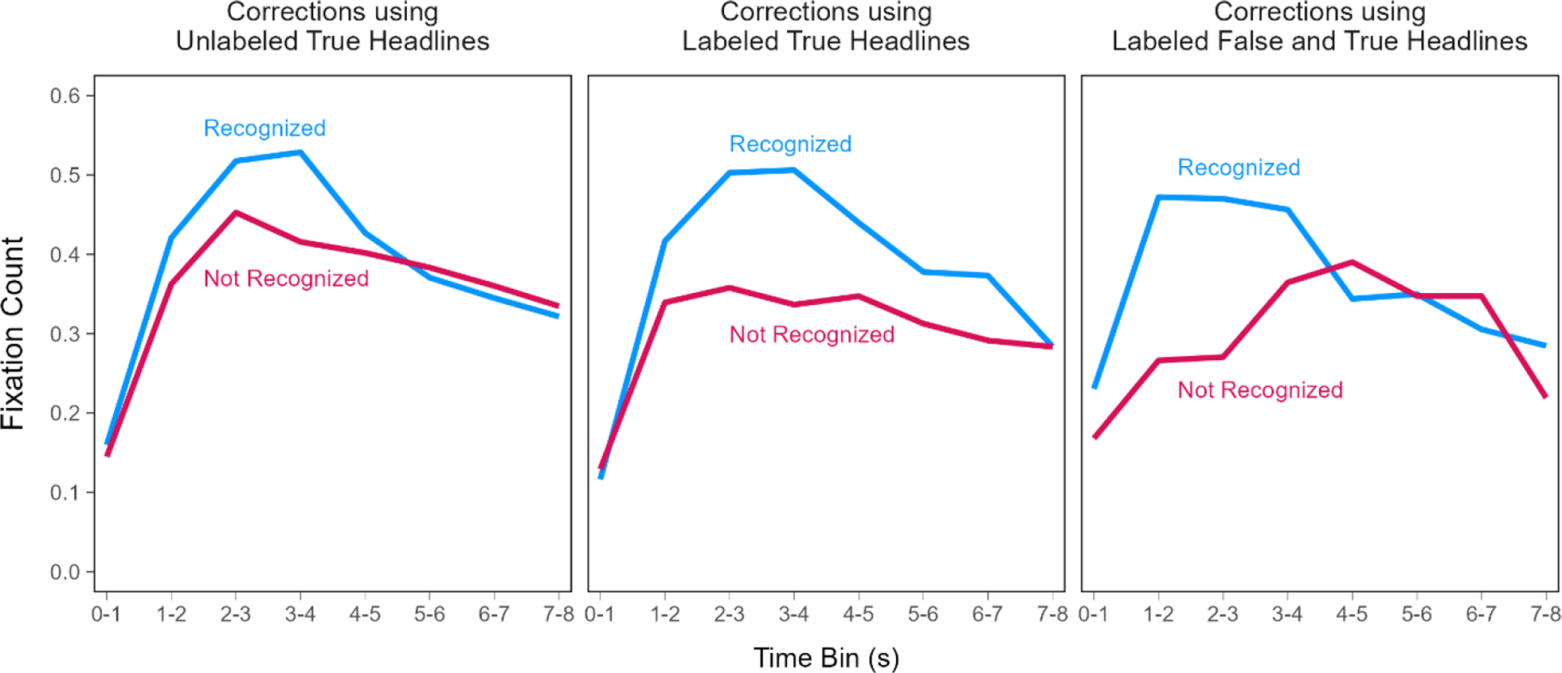
Fixations on key details in the correction phase conditioned on correction recognition in the re-evaluation phase. *Note*: Number of fixations when corrections were recognized (blue lines) and not recognized (red lines). The lines connect model population estimates at each time bin

To more fully characterize the association between fixation timing and correction recognition, we examined the time course of fixation counts in the correction phase conditioned on correction recognition (Fig. 11) using the same 1-s binning approach as before. Table 2 displays the results from a model with fixed effects of headline type, time bin, and correction recognition [*R*_*m*_^2^ = 0.02, *R*_*c*_^2^ = 0.16]. We do not elaborate on effects redundant with those reported above. The model indicated significant effects of headline type, time bin, correction recognition, smallest χ^2^(2) = 13.31, *p* = 0.001, and significant two-way interactions of time bin with headline type and correction recognition, smallest χ^2^(7) = 32.81, *p* < 0.001, *OP* = 99%. There was no significant two-way interaction with headline type and correction recognition, χ^2^(2) = 5.81, *p* = 0.055, and no significant three-way interaction, χ^2^(14) = 21.02, *p* = 0.101, *OP* = 85%. The time bin by correction recognition interaction showed that more fixations accumulated across approximately the first half of the viewing period for corrections that were subsequently recognized as compared to those that were not recognized.

**Table 2:** Model results for fixation counts across correction headlines and time bins conditioned on correction recognition

Effect	χ^2^	*df*	*p*
Headline type	13.31	2	= .001
Time bin	564.44	7	< .001
Correction recognition	28.64	1	< .001
Headline type × time bin	33.72	14	= .002
Headline type × correction recognition	5.81	2	= .055
Time bin × correction recognition	32.81	7	< .001
Headline type × time bin × correction recognition	21.02	14	= .101

**Fig. 12:**
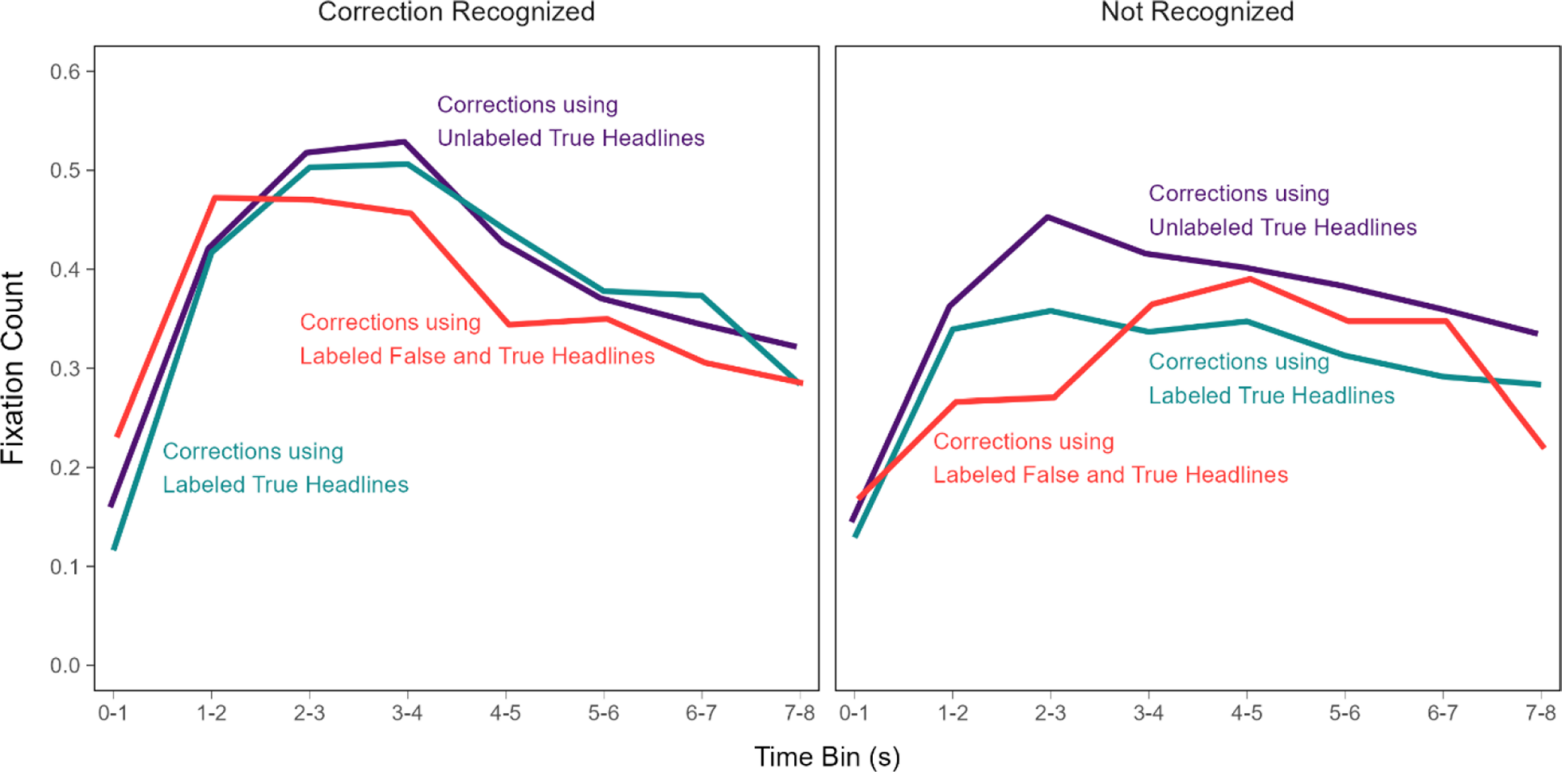
Fixations on key details in the correction phase conditioned on correction recognition in the re-evaluation phase over time. *Note*: Number of fixations when corrections were recognized (left panel) and not recognized (right panel). The lines connect model population estimates at each time bin

Although the three-way interaction was not significant, the patterns in Fig. 11 suggest that the distinct time courses that depended on correction recognition may have varied across headline types. We examined this further with exploratory analyses. The reader should therefore interpret the following results with appropriate caution. We modeled the time courses separately for corrections that were versus were not recognized. Figure 12 re-plots the time courses from Fig. 11 to highlight the contrasts across headline types within levels of correction recognition.

The model for recognized corrections [*R*_*m*_^2^ = 0.02, *R*_*c*_^2^ = 0.18] (Fig. 12, left) indicated no significant effect of headline type, χ^2^(2) = 2.53, *p* = 0.282, a significant effect of time bin, χ^2^(7) = 475.16, *p* < 0.001, and a significant interaction, χ^2^(14) = 39.55, *p* < 0.001, *OP* = 98%. Pairwise comparisons showed that fixation counts for corrections using labeled false and true headlines (red line) were significantly greater than for the other corrections (purple and green lines) at the 0–1 s bin, smallest z ratio = 2.37, *p* = 0.047, *d* = 0*.*11, and significantly less than for the other corrections at the 4–5 s bin, smallest z ratio = 2.41, *p* = 0.042, *d* = 0*.*11. The differences between the other corrections in those bins were not significant, largest z ratio = 1.12, *p* = 0.505, *d* = 0*.*05. There were no significant differences within the other bins, largest z ratio = 2.17, *p* = 0.076, *d* = 0*.*10. In contrast, the model for unrecognized corrections [*R*_*m*_^2^ = 0.01, *R*_*c*_^2^ = 0.14] (Fig. 12, right) indicated a significant effect of time bin, χ^2^(7) = 118.17, *p* < 0.001, *OP* = 100%. There was no significant effect of headline type, χ^2^(2) = 5.04, *p* = 0.080, and no interaction, χ^2^(14) = 15.90, *p* = 0.319, *OP* = 72%. These results converge with the prior findings showing that fixating earlier and more often to corrective details is associated with correction recognition. Moreover, these results uniquely showed that reminder-based corrections increased this earlier-looking tendency.

#### Fixations on key details in the correction phase predicting correction recognition in the re-evaluation phase

**Fig. 13:**
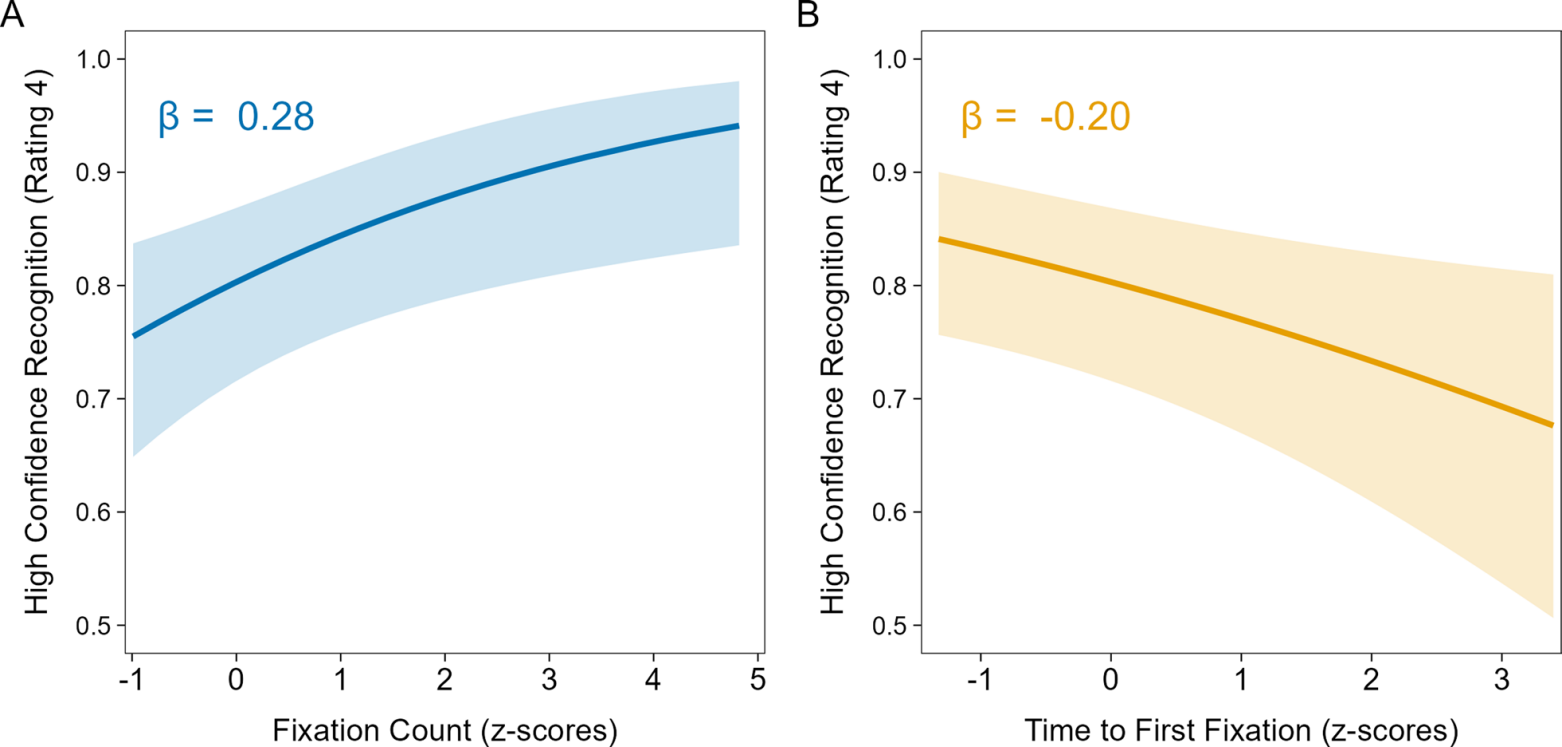
Predicted probabilities of correction recognition based on correction phase fixation measures. *Note*: Predicted probabilities of participants giving the highest rating (4, definitely corrected) when indicating their memory for corrections (i.e., correction recognition) based on the number of fixations (**A**) and time to first fixation (**B**) on key details of true headlines in the correction phase (Phase 2)

To complement the subsequent memory analyses of associations between fixations and correction recognition, we also conducted predictive analyses, using standardized fixation count and time to first fixation to predict correction recognition (Fig. 13). To determine the simplest model specification, we started with two separate models including headline type and either fixation counts [*R*_*m*_^2^ = 0.07, *R*_*c*_^2^ = 0.49] or time to first fixation [*R*_*m*_^2^ = 0.06, *R*_*c*_^2^ = 0.51] as fixed effects with correction recognition as the outcome variable. Each model also included initial evaluation phase veracity ratings as a covariate to control for differences in the encoding of corrections based on preexisting beliefs. Both models revealed significant effects of headline type, smallest χ^2^(2) = 46.62, *p* < 0.001, *OP* = 100%, and their respective fixation variables, smallest χ^2^(1) = 7.56, *p* = 0.006, *OP* = 20%. Importantly, both models revealed no significant interaction, largest χ^2^(2) = 1.43, *p* = 0.490, *OP* = 16%, suggesting that headline type could be dropped.

A reduced model including both standardized fixation predictors as continuous fixed effects [*R*_*m*_^2^ = 0.03, *R*_*c*_^2^ = 0.47], thus controlling for each other, along with initial evaluation phase veracity ratings as a covariate showed that fixation counts positively predicted correction recognition, *β* = 0.28, SE = 0.11, *p* = 0.008, *OP* = 84%, whereas time to first fixation negatively predicted correction recognition, *β* = − 0.20, SE = 0.08, *p* = 0.016, *OP* = 64%. (Because fixation counts and time to first fixation were measured as continuous variables, we estimated *OP* using a z-test.) These associations suggest the overall number of fixations on key details following corrections and the timing of these fixations independently predicted correction recognition, but the number of fixations contributed more. These findings converge with the subsequent memory analyses in showing that more and earlier fixations on corrective true details in the correction phase each uniquely predicted high confidence correction recognition in the re-evaluation phase.

## Discussion

The present experiment examined the role of memory-guided attention in the benefits of reminder-based corrections for updating beliefs in false headlines and remembering that false headlines were corrected. Replicating prior findings, reminding people of false headlines before presenting true headlines with corrective details reduced the perceived veracity of false headline more than presenting true headlines without reminders (Wahlheim et al., 2025). Reminder-based corrections also promoted the best memory for corrections. For all correction formats, the reduction in perceived veracity of false headlines was greater for recognized than unrecognized corrections. Together, these results suggest that reminder-based corrections improved the accuracy of beliefs partly by improving memory for corrections. Eye fixations suggested this subsequent memory effect could be attributed to memory-guided attention to corrective details improving encoding of those details. While fixation counts to true details did not differ among correction formats within any phase, reminder-based corrections directed fixations to true details earliest in the correction phase. Participants also allocated more fixations and looked earlier to true details for topics that were subsequently recognized as corrections, which occurred most for reminder-based corrections. Higher counts and earlier onset of such fixations also predicted correction recognition, even when both predictors were controlled for each other and when controlling for baseline beliefs. These findings suggest that repeating false details directed attention to true details, thus improving memory for corrections upon which beliefs were based.

The present findings are relevant for theories of misinformation correction effects on belief updating. Two theories make competing predictions about the effects of repeating misinformation on correction efficacy. Repetition-induced truth accounts, including the familiarity backfire account, predict that repeating misinformation can undermine correction efficacy by increasing misinformation familiarity, which is misattributed as veracity (Hasher et al., 1977; Skurnik et al., 2005). Conversely, the integrative encoding account predicts that reminders will enhance correction efficacy by promoting memory for associations between false and true information (Ecker et al., 2017; Kendeou et al., 2014; Wahlheim et al., 2020). The present finding that reminder-based corrections reduced the perceived veracity of false headlines the most is more compatible with integrative encoding than repetition-induced truth accounts.

Additional evidence for integrative encoding was shown by reminders leading to the most frequent high confidence recognition of corrections, which was associated with the bulk of the reduction in false beliefs. These patterns are similar to results from earlier studies showing enhanced correction efficacy when false information was repeated before true information, which was attributed to integrative encoding (Ecker et al., 2017; Kemp et al., 2022a, 2022b; Wahlheim et al., 2020, 2025). However, one caveat here is that ratings of veracity and memory for corrections could have been based on memory for the time that headlines appeared, which was twice as long for reminder-based than other corrections. It is certainly plausible that longer exposure improved memory for corrections independent of the looking evoked by reminders. While the present findings cannot fully eliminate this possibility, early looking to true details—which was greatest for reminder-based corrections—also predicted correction recognition for corrections without reminders of false information. Collectively, these findings suggests that the attentional guidance to true details conferred by reminders of false details uniquely improved correction recognition, but the magnitude of such effects could have been slightly overestimated here. More precise estimation of such reminder effects could be obtained in a future experiment that equates exposure time across correction formats.

The effects of integrative encoding, memory for correction, and belief updating are supported by broader literature showing a role for memory in belief updating (Swire-Thompson et al., 2023; Wahlheim et al., 2025). In those studies, the extent to which participants reduced their beliefs in misinformation was associated with their memory for corrections. When false beliefs regressed toward baseline levels over time, such regression was associated with the extent to which participants showed poorer memory for corrections. Those studies did not support the familiarity backfire prediction as false beliefs did not exceed baseline levels following reminder-based corrections. The present experiment was not ideal for testing the familiarity backfire account because it did not collect perceived veracity ratings after a longer delay. However, it still showed that belief updating depended on memory for corrections, as in earlier studies.

The unique contribution of the present experiment is the evidence suggesting that reminders enhanced memory for corrections, which serves as a basis for belief updating, partly by upregulating attention to and encoding of true details. More attentive encoding improves subsequent memory (for reviews, see Long et al., 2018; Sherman & Turk-Browne, 2022). Consistent with this empirical regularity, we showed that the increase in correction recognition following reminder-based corrections was associated with upstream attentional prioritization of true details. Specifically, viewers fixated on true details the earliest following reminders of false headlines. Viewers also fixated on true details more often (and earlier) for corrections that were remembered with the highest confidence, which occurred most after reminder-based corrections. Both the count and onset timing of fixations on true details uniquely predicted correction recognition, with increased, early fixations corresponding to the best memory outcomes. Together, these findings indicate that reminder-based corrections upregulated attention to details indicative of headline veracity more effectively than other correction methods. Consistent with a prior study of memory updating for actions (Wahlheim et al., 2022), these findings suggest that enhanced attentional focus engendered more opportunities to compare false and true details, thus supporting integrative encoding and subsequent memory for those details and their veracity.

Other misinformation studies have shown a critical role for attention in how people discern the truth of headlines. For example, truth discernment for social media posts has been shown to benefit from directing attention to the accuracy of content before engaging with the posts (Pennycook et al., 2020, 2021; Pennycook & Rand, 2022; but see Ceylan et al., 2023; Roozenbeek et al., 2021). Evidence from the information systems literature also demonstrates that asking social media users to rate news veracity reduces susceptibility to misinformation (e.g., Moravec et al., 2022). These findings suggest that task goals provide top-down guidance of attention to the features of posts that inform viewers’ assessments of information veracity. Eye tracking studies have shown that increased attention to true details is associated with decreased perceived credibility for false information and increased memory for true information (for a review, see Kim et al., 2025). For example, health warning labels that received earlier first fixations and longer dwell times were recalled better, presumably because they held attention better during encoding (Strasser et al., 2012). Similarly, for misinformation corrections, corrective details that received longer fixation durations were associated with reductions in the credibility and perceived veracity of false information in social media posts (Kim et al., 2021). These studies converge in suggesting that provoking viewers to attend to details indicative of information veracity can improve belief and memory accuracy.

Previous eye tracking studies have also examined viewer looking behavior in social media contexts, but few have identified how specific features of corrections influence how viewers direct attention. These studies have primarily focused on attention to components of social media posts and have rarely included attention to veracity. For example, fixation measures have shown that people attend to the source and comments of news posts more when considering if they would read the full article (Sülflow et al., 2019). Dwell time has shown that social media posts with additional content in the form of pictures and links automatically capture more attention (Vraga et al., 2016). These studies show that the structure of social media posts influences how viewers allocate attention. Other work has shown that correction formats can be varied to better capture attention. For example, correction posts that included humorous images were viewed more than non-humorous correction posts, and this was associated with reduced false beliefs (Kim et al., 2021). These findings suggest that individual features of corrections on social media can be optimized to capture attention and improve correction efficacy.

Our study extends this literature by constraining viewing options to reveal how the structure of perceptual inputs guides attention, with or without viewers having control. Here, reminders may have regulated attention by compelling viewers to intentionally look for true details to compare to recently viewed false details. Additionally, exposure to reminders might have facilitated more automatic attentional capture to the change in a small set of perceptual features (i.e., the characters that changed in the areas of interest). Detection of perceptual changes between true and false headline versions may have triggered an upregulation of attention that preceded conceptual processing that occurred when perceived true details were co-activated with memories of false details, thus enabling integrative encoding. This alternative perspective does not rule out the integration account, but it does offer a nuanced view on the relative contributions of various component processes that may lead to reminder-based correction benefits. We did not evaluate viewer control over attention, and the present results do not indicate the extent to which reminders regulated attention through top-down or bottom-up processing. Future research could query viewers’ metacognition about how they direct their attention while encoding, which could inform whether reminder-based corrections help strategically guide attention in real-world social media settings.

The present findings that reminders guide attention to corrective details and improve belief updating suggest that, in applied contexts, misinformation should appear in formats that verifiably increase attention to true details. This technique can be applied on social media by flagging false information with the suggestion that viewers should click through to read the true details. Presenting labeled misinformation and a corrective statement simultaneously may also be an appealing option, such as when social media posts with false information are shown with corrective community comments. However, correction formats should also consider how the order in which true and false information is displayed might impact correction efficacy (Vraga et al., 2020). Because the present findings identify attention to and memory for false and true information as critical for belief updating, correction formats should make it a priority to capture and direct attention to differences in that information. This is especially important given that divided attention while viewing social media can impair belief accuracy (e.g., Pennycook et al., 2020). Designers of misinformation correction techniques should therefore first verify the most effective timing and placement of reminders for attracting and sustaining attention under distracting conditions.

## Limitations

The present study focused on the benefits of reminders on immediate belief updating and identified memory-guided attention as a key mechanism. However, it did not examine the extended downstream consequences of this improvement in attention over time. Additional research could include a second post-correction test after a delay to examine if the belief regression is also associated with fixations to corrective details. Research has shown that corrections with false before true information maintain the lower false beliefs than corrections with only false or only true information (Wahlheim et al., 2025). Eye tracking could be used to determine the role of attention in sustained belief change that occurs when reminders promote integrative encoding.

The study materials included politically neutral headline topics to limit partisanship bias. Because topics that were initially neutral can become politicized over time, it is impossible to fully control for political thinking in our experiment. We did not measure participant partisanship or the extent to which headlines evoked political thinking. We counterbalanced our materials and controlled for item-specific effects in statistical models to limit these influences. However, partisanship may have influenced headline evaluation for some topics. Future studies could explicitly account for effects of political thinking and participants’ political partisanship by collecting those ratings.

Our study examined the benefits of reminder-based corrections in a social media context. However, to bring key variables under experimental control, we designed a task that does not generalize perfectly to social media sites. We limited exposure to headlines and corrective elements to a fixed duration rather than allowing participants to control the amount of exposure. Headlines encountered on social media may also contain more elaborative changes in language from original to correction posts and more ambiguities in language than the headlines used in our study. Users also experience distractions when viewing corrections on social media, including unrelated posts and advertisements. These distractors may limit the efficacy of reminder-based corrections when they limit attention to veracity details (e.g., Pennycook et al., 2020). Future research could aim to extend our approach to a context that more closely reflects the user experience on social media. An experiment of this sort could allow for free viewing of headline corrections and include distractor elements such as unrelated content, advertisements, and the ability to interact with posts through likes and sharing. This could inform how users regulate their attention while using social media in a realistic context and how the benefits of reminder-based corrections can be enhanced or diminished when these factors are included.

Mimicking the user experience on social media also necessitated greater exposure time for reminder-based corrections in the present experiment. We did not increase exposure time for other corrections to avoid excessive exposure to corrections that our pilot work showed could be read in the time allotted here. Although our results suggest that reminders improved correction memory independent of exposure time, future work should empirically test this by equating exposure time across correction methods. This could be accomplished by increasing the duration of corrections with veracity labels to match the duration of reminder and subsequent corrections or by simply presenting corrections with veracity labels twice in a row. Either approach would inform the extent to which exposure time contributes to the memory benefits associated with reminders but with a cost to the ecological validity and practicality of the procedure.

Our study used recognition to evaluate memory for corrections. A final limitation is that we did not use additional approaches to assess memory for both corrections and the headline content itself, such recall of true and false details. Recall of headline content could inform whether reminder-based corrections improve memory for true information without repeating headline wording as part of retrieval cues. Other studies have shown the mnemonic benefits of reminders using similar paradigms with a cued recall final test (Kemp et al., 2022a, 2022b, 2024a, 2024b; Wahlheim et al., 2020). Future work could extend these findings by replicating reminder benefits with cued recall paradigms and assessing fixation pattern-to-recall associations.

## Conclusion

The present study provided evidence for the role of memory-guided attention in the beneficial effects of reminder-based corrections on beliefs and memory. Reminder-based corrections reduced false beliefs more than corrections with only true information. These benefits were associated with earlier fixations to true corrective details. Viewers were also more likely to confidently recognize corrections that were preceded by more fixations on corrective details. Our results suggest that reminders upregulate attention to true details and enhance conflict saliency to improve belief updating, consistent with the integrative encoding account, though future work will require controlling exposure durations to more stringently test this account. These conclusions are supported by broader literature showing that memory for corrections is critical in belief updating and that attention during encoding determines whether information is remembered. Collectively, these findings show that memory-guided attention plays a critical role in updating beliefs to guard against the negative consequences of misinformation exposure. Based on this information, correction methods may benefit from spatiotemporal design features that direct and sustain attention to the contrast between false and true information. 

## Data Availability

The datasets supporting the conclusions of this article are available in the Open Science Framework repository, https://osf.io/jfvea/. The materials, data, and analysis scripts are available on the Open Science Framework: https://osf.io/jfvea/. The experiment was not preregistered.

## Declarations

### **Significance statement **

Information shared on social media can reach up to millions of users almost instantaneously. An unintended consequence of this rapid communication is an unprecedented spread of misinformation. Exposure to misinformation online has serious real-world consequences. For example, believing false information about viruses and politicians may influence health guideline compliance and voter behavior. Social media platforms have tried to address this problem by issuing misinformation corrections. The present experiment helps us understand the cognitive processes, namely attention guided by memory, that contribute to the success of corrections that use misinformation reminders. Reminding people of misinformation before correcting it can be effective in promoting accurate beliefs and memories. This may partly be because repeating false details before correcting them can draw attention to contrasting true details in a correction, leading to improved memory for both. We found those benefits here and, using eye tracking, showed that earlier and more attention to true details following reminders predicted these benefits. Our findings suggest that reminder-based corrections mitigate misinformation exposure partly because they direct attention to true details, thus supporting memories upon which people can base their beliefs. An everyday implication for social media platforms is that corrections may best promote attention and memory when their features contrast true and false information.
